# Automated planning through robust templates and multicriterial optimization for lung VMAT SBRT of lung lesions

**DOI:** 10.1002/acm2.12872

**Published:** 2020-04-10

**Authors:** Livia Marrazzo, Chiara Arilli, Roberto Pellegrini, Pierluigi Bonomo, Silvia Calusi, Cinzia Talamonti, Marta Casati, Antonella Compagnucci, Lorenzo Livi, Stefania Pallotta

**Affiliations:** ^1^ Careggi University Hospital, Medical Physic Unit Florence Italy; ^2^ Elekta AB Stockholm Sweden; ^3^ Careggi University Hospital, Radiotherapy Unit Florence Italy; ^4^ Department of Experimental and Clinical Biomedical Sciences “Mario Serio” University of Florence Florence Italy

**Keywords:** automated planning, lung SBRT, robust templates, VMAT

## Abstract

**Purpose:**

To develop and validate a robust template for VMAT SBRT of lung lesions, using the multicriterial optimization (MCO) of a commercial treatment planning system.

**Methods:**

The template was established and refined on 10 lung SBRT patients planned for 55 Gy/5 fr. To improve gradient and conformity a ring structure around the planning target volume (PTV) was set in the list of objectives. Ideal fluence optimization was conducted giving priority to organs at risk (OARs) and using the MCO, which further pushes OARs doses. Segmentation was conducted giving priority to PTV coverage. Two different templates were produced with different degrees of modulation, by setting the Fluence Smoothing parameter to Medium (MFS) and High (HFS).

Each template was applied on 20 further patients. Automatic and manual plans were compared in terms of dosimetric parameters, delivery time, and complexity. Statistical significance of differences was evaluated using paired two‐sided Wilcoxon signed‐rank test.

**Results:**

No statistically significant differences in PTV coverage and maximum dose were observed, while an improvement was observed in gradient and conformity. A general improvement in dose to OARs was seen, which resulted to be significant for chest wall V_30 Gy_, total lung V_20 Gy_, and spinal cord D_0.1 cc_. MFS plans are characterized by a higher modulation and longer delivery time than manual plans. HFS plans have a modulation and a delivery time comparable to manual plans, but still present an advantage in terms of gradient.

**Conclusion:**

The automation of the planning process for lung SBRT using robust templates and MCO was demonstrated to be feasible and more efficient.

## 1 INTRODUCTION

The planning and quality assurance (QA) required for intensity‐modulated radiation therapy (IMRT) and volumetric modulated arc therapy (VMAT) are more complex and time consuming compared with conventional conformal radiotherapy (CRT) techniques, which can have significant impact on the resources of a radiotherapy department.^1^

Besides constituting a huge workload, manual planning is considerably dependent on planner experience and available planning time, thus producing large variations in plan quality.^2^

Quite recently, automatic planning was proposed as an option in several treatment planning systems (TPS), with different approaches, with the purpose of decreasing the time required for planning and the interoperator heterogeneities.^3^, ^4^, ^5^, ^6^ A common finding of several studies investigating the effect of automatic planning was the increase in planning efficiency and consistency when these tools are introduced.^7^, ^8^, ^9^, ^10^

Nevertheless, the commercial automatic planning systems are not available in all radiotherapy departments, since they are proprietary systems, requiring specific licenses. Being able to automate the planning process through the use of the available tools would constitute a large advantage, making eventually exportable the developed automated technique to different centers that make use of the same TPS.

There is interest, for example, in robust templates, which are applicable to a wide range of cases (a class solution) producing good plans in a large number of patients with no/low manual intervention. A class solution is defined as a set of planning objectives, penalty parameters, and beam arrangements that are robust enough to produce a clinically acceptable dose distribution regardless of patient size, anatomy, target volumes, and organs at risk (OAR).^11^

The possibility of introducing class solutions, with the aim of improving consistency in plan quality and efficiency in planning, has been already explored and there are several publications on this topic. To our knowledge, the only published experience on lung planning (no SBRT) was from Bral and colleagues,^12^ who developed a robust template for treatment of stage III nonsmall cell lung cancer using helical TomoTherapy (70.5 Gy in 30 fractions) with fixed constraints and priorities. Their class solution resulted in a deliverable plan in all 40 consecutive patients considered in the study.

Some work has been done in developing class solutions for the treatment of prostate with IMRT,^11^, ^13^ even in case of particularly challenging anatomical situations, as in the case of bilateral hip prostheses,^14^ and VMAT, for both conventional fractionation^15^, ^16^ and SBRT.^17^, ^18^ These studies demonstrated automated planning for prostate cancer is feasible and can produce plans that are at least as good as manual plans. Moreover, the development of class solutions allows for the “bias‐free” comparison between different techniques by applying the previously established templates.^15^

Class solutions have been used for technique comparison in breast treatment,^19^, ^20^ while a five‐field IMRT template was established and evaluated on 40 patients treated with accelerated partial breast irradiation for left and right lesions.^21^ The developed template was demonstrated to produce highly conformal and consistent treatment plans.

An improvement in planning efficiency (planning reduction time by 30–60%) was proved in the work of Weksberg et al.,^22^ where the class solution was developed for spinal SBRT and applied to 91 patients.

A common conclusion of the above cited studies is that a properly built class solution can result in deliverable plans in large cohorts of patients.

In the present report, we describe the development of a class solution for patients treated with lung SBRT (55 Gy in 5 fractions) using VMAT and the Monaco treatment planning system (Elekta AB, Sweden). The element of novelty, apart from the fact that it is the first published experience on lung SBRT, lies in the coupling of the robust template with the multicriterial optimization (MCO) available in Monaco. The robust template produces plans that are reproducible among patients, giving consistent results (thus enhancing standardization), while the use of the MCO is able to effectively account for anatomical changes, thus producing personalized results.

In our Institute around 100 patients are treated per year with lung SBRT. Therefore, besides improving consistency and quality of planning, development of an effective robust template for this frequent treatment has the potential to significantly impact efficiency.

## 2 MATERIALS AND METHODS

### 2.A Patients and planning

All patients in this retrospective study were planned with the Monaco planning system (version 5.10, Elekta AB, Stockholm, Sweden) which uses a Monte Carlo algorithm for dose calculation. A Synergy linac (Elekta AB, Stockholm, Sweden) equipped with a Beam Modulator (21 × 16 cm^2^ maximum field size, 0.4 cm leaf width at isocenter) and VMAT delivery was used for all plans.

Patients were immobilized using an Extended Wing Board™ (Civco Radiotherapy, Iowa, US) and imaged on a Philips Big Bore CT (Philips Medical Systems, Cleveland, OH, USA) with a 4D‐CT acquisition. The external respiratory signal was used for phase sorting; it was collected with a Philips Bellow system (Philips Medical Systems, Cleveland, OH, USA). Data were acquired in helical mode with 3 mm slice thickness and by selecting an optimal pitch depending on the patient's respiratory frequency.

Gross tumor volume (GTV) was contoured on each of the 10 reconstructed phases and an internal target volume (ITV) was then built as an envelope of GTV contours. Planning target volume (PTV) was obtained with a 5‐mm isotropic expansion around the ITV. Average CT was used for planning. In addition, the following OARs were contoured: lungs, heart, esophagus, spinal cord, and chest wall.

The characteristics of patient sample used for template validation are reported in Table 1. A large variability is present in the patient sample, both in treatment volume and location, useful for assessing the general validity of the template.

**TABLE 1:** Characteristics of patients in the validation set.

Gender	
Male	12
Female	8
Age (years) (range)	66 (38–88)
Diagnosed primary tumor	
Nonsmall cell lung cancer	12
Sarcoma	3
Breast	2
Kidney	2
Colon	1
Tumor location	
Left lung	9
Superior lobe	7
Inferior lobe	2
Right lung	11
Superior lobe	4
Central lobe	6
Inferior lobe	1
Directional tumor position	
Posterior	9
Lateral	6
Anterior	5
Average PTV volume (cc) (range)	30 ± 20 (8.3–77.1)

PTV, planning target volume.

Manual clinical plans were generated by five different experienced planners. In general, arcs amplitudes, planning objectives, and other planning parameters (such as the use of a ring ROI around the PTV to improve conformity) were differently chosen based on planner preferences. MCO was also eventually used on some OARs cost functions at the discretion of the planner.

Requirements on PTV coverage and OAR sparing are summarized in Table 2. They are derived from the UK consensus Guidelines.^23^

**TABLE 2:** PTV and OARs requirements for the lung SBRT VMAT plans in five fractions.

Organ	Constraint	Optimal	Mandatory
PTV	V95%	100%	≥98%
	D_max_ (0.1 cm^3^)	≤60 Gy	–
Spinal canal (including medulla)	D_max_ (0.1 cm^3^)	<23 Gy	<30Gy
	D_1 cm_^3^	<14.5 Gy	–
Esophagus	D_max_ (0.5 cm^3^)	<32 Gy	<34 Gy
Heart	D_max_ (0.5 cm^3^)	<27 Gy	<29 Gy
Chest wall	D_30 cm_^3^	<32 Gy	–
Normal lungsm(lungs – gross tumor volume)	V_20 Gy_	–	<10%

PTV, planning target volume; OARs, organs at risk; V_XGy_, volume receiving X Gy; D_Xcm_^3^, dose to X cm^3^ of the considered organ.

### 2.B Robust template construction and validation

**FIG. 1:**
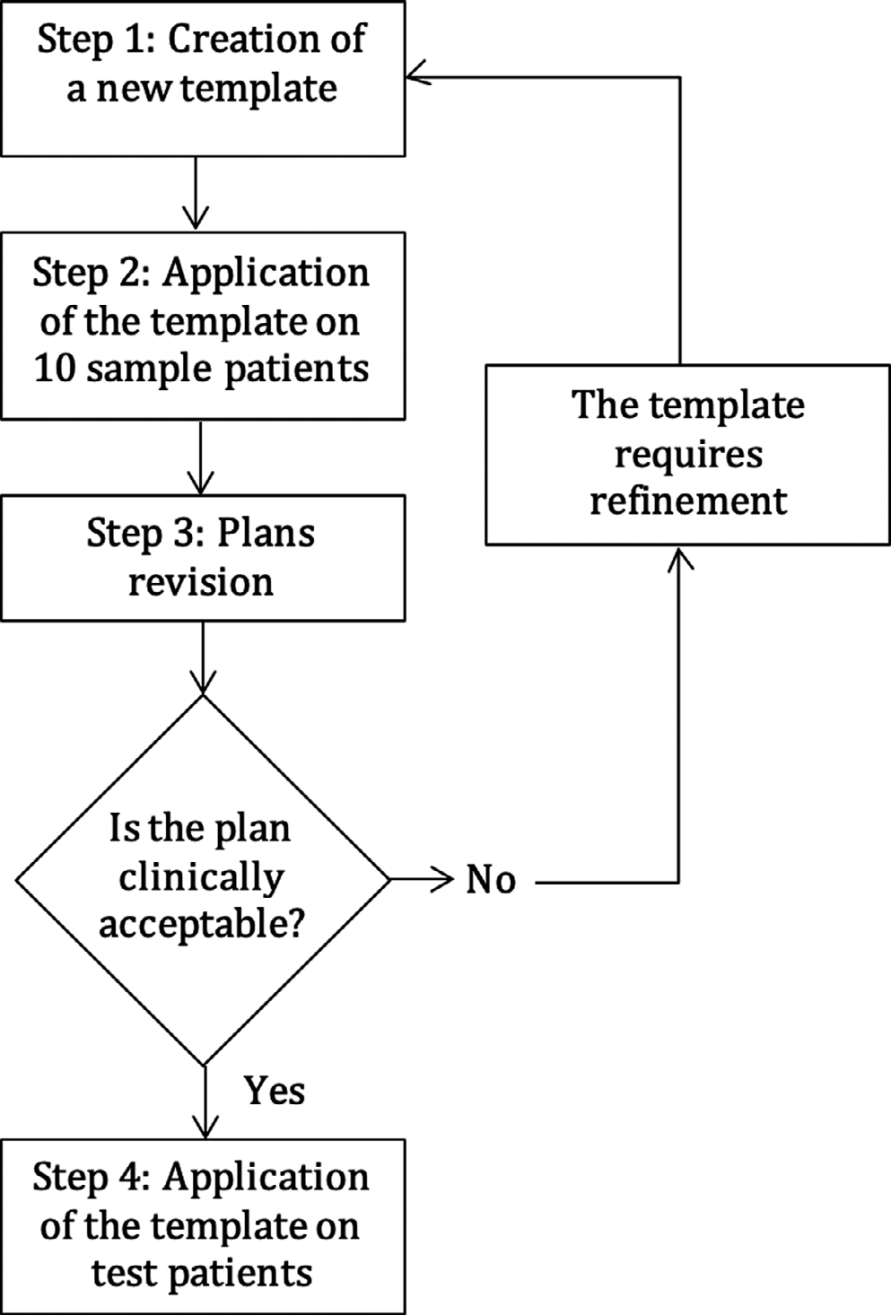
Flowchart of the stepwise quality improvement model for the development of a class solution for lung SBRT VMAT. A template is created, applied to the first patient of the sample and modified until the produced plan is clinically acceptable. Then the template is applied to the second sample patient: if the resulted plan is clinically acceptable, then the template is applied to further patients. Otherwise it is modified as requested and then applied to further patients (including the first). Mainly the template is optimized by iteratively changing the cost functions or the isoconstraints.

The template, containing geometrical and planning settings and a list of planning objectives, was first established on a population of 10 lung SBRT patients planned for 55 Gy in 5 fractions (peripheral lesions, near to or partly overlapped with the thoracic wall) and refined with a stepwise process (Fig. 1). To improve gradient and conformity a 4‐cm isotropic expansion of the PTV (named ring) was created and set in the list of objectives. Conversely to what is commonly done in SBRT, the prescription isodose line is high (about 91%) and D_max_ (0.1 cm^3^) is kept below 60 Gy (Table 2). This choice is done due to the proximity or overlapping of the PTV with the chest wall, for reducing toxicity (chest wall pain and rib fractures).

In order to account for anatomical differences among patients, so to achieve personalized results, stage 1 (ideal fluence optimization) was conducted giving priority to OARs (Constrained mode) and using the MCO option of Monaco for all the OARs cost functions. MCO further pushes OARs dose, stopping just before compromising target coverage.

In Monaco the term “isoconstraint” is used to indicate what we asked the system for, while the term “isoeffect” indicates the observed result. Constrained mode ensures that the constraints on the OARs are always met, meaning that the run‐time isoeffects of each different cost function achieves a lower value than the isoconstraint. Then the objectives of PTVs coverage are possibly achieved by the optimizer. For the cost functions that have the MCO, when both OARs constraints and PTV objectives are satisfied, then the system tries to achieve even lower isoeffects on those cost functions of the OARs until a further reduction starts to affect the PTVs coverage.^24^

Stage 2 (segmentation) was conducted giving priority to PTV coverage (Pareto mode).

The template was built with two coplanar ipsilateral partial arcs (205°) and it contains objectives on PTV, ring, and patient, used to achieve target coverage, gradient, and conformity and to limit the maximum PTV dose. Since we want the template to be as general as possible, we also included spinal cord, esophagus, heart, and total lungs as OARs, independently of their proximity to the target. The dose to the thoracic wall was controlled and achieved by the ring cost functions.

Since the MCO option tends to produce plans with a high modulation, two different “fluence smoothing” options were explored: medium and high (producing the plans defined as MFS and HFS, respectively).

After the refinement phase, the final template was applied (with no manual intervention) on 20 further patients and the resulting plans were compared with the manual clinical plan.

### 2.C Dose distribution comparison

Dose distributions were compared in terms of dosimetric plan parameters (dose to PTV, conformity and gradient and dose to OARs). Conformity was measured by means of Paddick Conformity Index (PCI)^25^ that is defined as:

(1) $$\text{PCI}=\frac{{\text{TV}}_{\text{PIV}}^{2}}{\text{TV}\times \text{PIV}}$$

where TV_PIV_ is the volume of the target covered by the prescription isodose, TV is the target volume, and PIV is the prescription isodose volume. Gradient was measured with the Gradient Index (GI),^25^ which is defined as the ratio of the volume of half the prescription isodose to the volume of the prescription isodose.

Plans were also compared in terms of delivery time, monitor units, and plan complexity (through the Modulation Complexity Score, MCS^26^). Due to its mathematical formulation, the MCS has values in the range from 0 to 1. MCS = 1 means no modulation and can be exemplified by an arc with a fixed rectangular aperture with no leaves moving during the delivery. As modulation increases, MCS decreases.

Statistical significance of differences between automatic and manual plans was evaluated using paired two‐sided Wilcoxon signed‐rank test with a significance level of 0.05. All statistical analyses were performed with OriginPro (version 9.0.0, OriginLab Corporation, Northampton, MA).

### 2.D Dosimetric verification

In order to assess whether the planned dose distributions could be reliably delivered, dosimetric verification was performed and evaluated in terms of γ passing rate (global, 3%/2 mm) and point dose measurements. Each patient plan was transferred to the ArcCheck^®^ (Sun Nuclear Corporation, Melbourne, FL) 3D array, using the quality QA feature of Monaco TPS. Additionally, point dose measurement was performed with an Exradin A1SL ionization chamber (Standard Imaging, Middleton, WI) placed in the ArcCheck^®^ central plug.

## 3 RESULTS

Cost functions and parameters used in the final template validated in this work are reported in Supplementary Table 1. The template file is also available online as Supplementary Material.

**FIG. 2:**
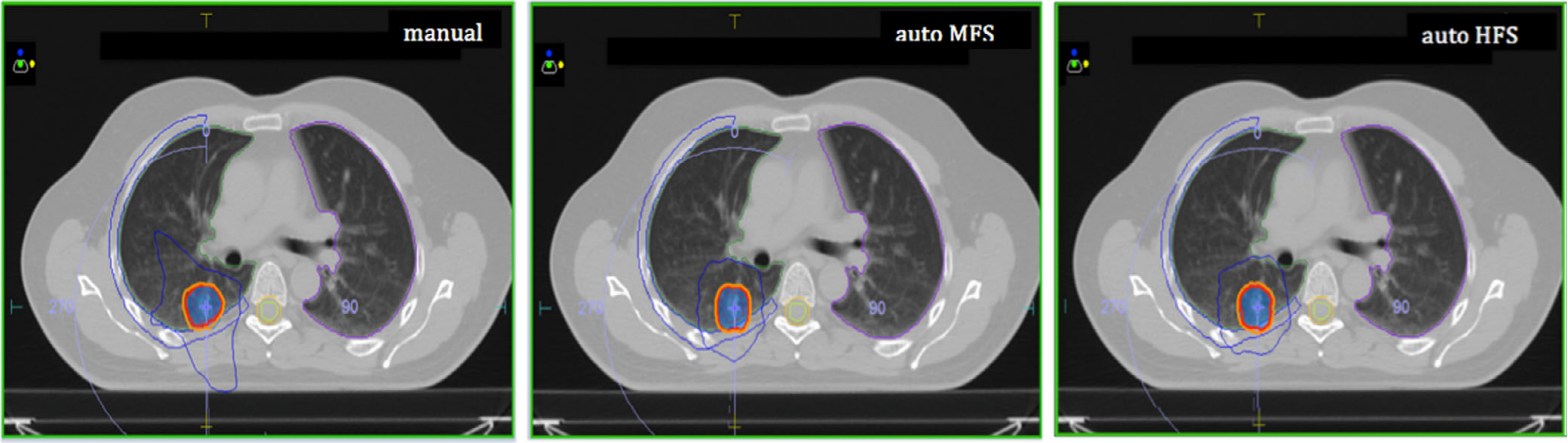
Comparison between manual, auto MFS, and auto HFS dose distributions. PTV is solid blue. The 100% prescription isodose is red, 95% prescription isodose is orange and 50% prescription isodose is blue. Fifty percent of prescription isodose reveals the large improvement in gradient in the automatic plans. MFS, Medium fluence smoothing; HFS, High fluence smoothing; PTV, planning target volume.

No statistically significant differences in PTV coverage and PTV maximum dose were observed (Table 3). PTV maximum doses (D_0.1cc_) ranges were: 56.3–60.5 Gy, 58.5–60.2 Gy, and 59.0–60.5 Gy for manual, MFS, and HFS plans, respectively. An improvement was observed in GI (statistically significant for both MFS and HFS plans) and PCI (statistically significant only for MFS plans). The improvement in gradient is due to the MCO effectively reducing the dose on the annular ring around the PTV. An example of dose distribution is shown in Fig. 2, where the shape of the 50% prescription isodose reveals the large improvement in gradient in automatic plans compared to manual.

**TABLE 3:** PTV and OARs parameters for MCO auto and clinical manual plans. In bold the P‐values expressing a statistically significant difference between auto and manual plan at the 0.05 level.

	PTV V_95%_ (%)	PTV D_0.1 cm_^3^ (Gy)	GI	PCI	Chest wall V_30 Gy_ (%)	Total lung V_20 Gy_ (%)	Heart D_0.5 cm_^3^ (Gy)	Esophagus D_0.5 cm_^3^ (Gy)	Cord D_0.1 cm_^3^ (Gy)	Cord D_1 cm_^3^ (Gy)
MCO MFS auto plan (AVG ± 1SD)	98.1 ± 1.2	59.5 ± 0.4	5.0 ± 0.6	0.83 ± 0.08	20.0 ± 12.3	5.2 ± 3.0	9.7 ± 7.6	8.7 ± 5.7	8.7 ± 4.5	8.0 ± 4.0
MCO HFS auto plan (AVG ± 1SD)	98.0 ± 2.0	59.6 ± 0.4	5.0 ± 0.7	0.75 ± 0.14	21.5 ± 12.5	5.4 ± 3.0	11.0 ± 8.1	9.5 ± 4.5	8.7 ± 4.3	7.8 ± 4.0
Clinical plan (AVG ± 1SD)	97.5 ± 1.9	58.7 ± 2.5	6.6 ± 1.4	0.77 ± 0.12	26.4 ± 14.0	6.2 ± 3.2	11.8 ± 9.3	10.4 ± 3.5	10.7 ± 4.3	9.1 ± 4.1
p MCO MFS vs clinical	0.3	0.1	**<0.01**	**0.02**	**<0.01**	**<0.01**	0.2	0.06	**<0.01**	0.07
p MCO HFS vs clinical	0.2	0.07	**<0.01**	0.7	**0.03**	**<0.01**	0.2	0.2	**<0.01**	**0.03**

MFS, Medium Fluence Smoothing; HFS, High Fluence Smoothing; OARs, organs at risk; PTV, planning target volume; V_95%_, volume receiving 95% of the prescription isodose; V_XGy_, volume receiving X Gy; D_Xcm_^3^, dose to X cm^3^ of the considered organ; GI, Gradient Index, volume encompassed by the 50% of the prescription isodose/prescription isodose volume; PCI, Paddick Conformity Index= (target volume in the prescription isodose)^2^/(target volume × prescription isodose volume).

A general improvement in dose to OARs was observed (Table 3), which resulted to be statistically significant for chest wall V_30 Gy_, total lung V_20 Gy_ and for cord D_0.1 cc_.

**FIG. 3:**
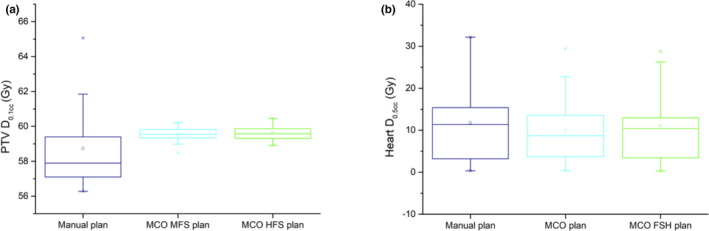
Box plots relative to PTV D_0.1cc_ (a) and Heart D_0.5cc_ (b) for manual, auto MFS, and auto HFS plans. MFS, medium fluence smoothing; HFS, high fluence smoothing; PTV, planning target volume.

Variability in target maximum dose (largely operator dependent in manual plans) and gradient index was reduced in the automated plans (standard deviations in Table 3). No reduction is observed in the standard deviations of OARs parameters, which are more dependent on anatomical heterogeneities than on interoperator variability. A representative example is shown in Fig. 3, where the box plots relative to PTV D_0.1 cc_ and Heart D_0.5 cc_ are reported.

Auto MFS are characterized by a significantly higher modulation and delivery time when compared to manual plans (Table 4), while the use of the high fluence smoothing option allows to obtain plans with a modulation complexity and a delivery time that are comparable to manual plans, without losing the advantage in gradient and dose to OARs.

**TABLE 4:** Plan complexity parameters and dosimetric verification results for MCO auto and clinical manual plans.

	MCS	Delivery time (s)	MU	γ passing rate (%) (3%/2 mm, global)	ΔD (%)
MCO MFS auto plan (AVG ± 1SD)	0.29 ± 0.07	448 ± 73	3379 ± 573	91 ± 3	−1.4 ± 1.4
MCO HFS auto plan (AVG ± 1SD)	0.40 ± 0.70	319 ± 41	2368 ± 294	96 ± 2	−0.2 ± 1.7
Clinical plan (AVG ± 1SD)	0.37 ± 0.13	297 ± 51	2146 ± 394	96 ± 3	0.9 ± 2.0
p MCO MFS vs clinical	**<0.01**	**<0.01**	**<0.01**	0.3	0.06
p MCO HFS vs clinical	0.7	0.2	**0.02**	1	0.59

MFS, Medium Fluence Smoothing; HFS, High Fluence Smoothing; volume; MCS, Modulation Complexity Score; MU, Monitor Units; ΔD, percentage dose difference. In bold the *P*‐values expressing a statistically significant difference between auto and manual plan at the 0.05 level.

Concerning dosimetric verifications, no statistically significant differences were observed in γ passing rates and point dose measurements (Table 4), although some degradation is observed for the more modulated MFS plans (average passing rate below 95%).

No manual plan changes were required: all the 20 plans automatically generated fulfilled the required constraints and were considered clinically acceptable by an experienced Radiation Oncologist. Average planning time was 8 ± 2 min, which was a large improvement if compared with about 120 min estimated for the manual planning.

## 4 DISCUSSION

In the present study, we explored the possibility of developing a robust template for automating the treatment planning process for lung SBRT. The template met treatment criteria in all patients and also resulted in an improvement when compared to manually generated plans, thus demonstrating that a properly built and validated robust template can be favorable compared to manual planning. The advantages (in terms of gradient and doses to OARs) were obtained thanks to the MCO option of Monaco TPS, which further pushes OARs dose, stopping just before compromising target coverage. This aspect constitutes one of the elements of novelty of our work, since optimizing in Constrained mode with MCO in the first step and then moving to Pareto mode (MCO off) during the segmentation step is a powerful way of working with Monaco, never published before, which enhances effectiveness of using templates. This approach does not need manual refinement of the plans, thus allowing to fully automate the planning process.

Even a small reduction in doses to OARs, although already below constraints, can be clinically significant in the light of patient retreatment.

We also observed an increase in treatment consistency. A reduction in plan variability (interoperator variability) was produced by automatic plans. This reduction is more evident on PTV than on OARs parameters, whose variations are more sensitive to anatomical heterogeneities than to interoperator variability.

A further advantage of building robust templates lies in the possibility of testing the differences produced by some planning settings, while leaving the same list of objectives. This is what we have done by changing the fluence smoothing parameter and demonstrating that sometimes the increase in plan modulation does not reflect in a sharp advantage on plan quality. Dose delivery with VMAT technique (where the dose modulation is obtained by the variation of leaves position and speed, gantry angle and speed, and dose rate) on moving targets could be affected by the so‐called interplay effect, which is caused by the interaction between dose delivery and anatomy movement.^27^ The quantification of the interplay effect in lung SBRT has been the subject of several papers, that demonstrated the interplay effect to be generally small, even for highly modulated intensity patterns.^27^ Nevertheless, with large motion and increased motion period^28^ or for respiratory curves exhibiting irregular breathing patterns^29^ the interplay effect may become significant, which supports the choice of a moderate modulation.

Our clinical choice of having a homogeneous dose distribution (using a high prescription isodose) also deserves some consideration, since it is an unusual choice in lung SBRT. Inhomogeneous dose distributions are generally considered to be advantageous for increasing the integral GTV dose and for OARs sparing.^30^ However, the PTVs of the patients' sample selected in our study are very near or partly overlapped to the chest wall, so the maximum dose was limited in order to reduce chest wall toxicity. A recently published pooled analysis of 57 studies,^31^ identified the maximum dose to chest wall (dose to 0.5 cm^3^ and 5 cm^3^) to be significantly associated with chest wall pain and rib fractures. Looking at the quality of our plans, gradient indexes are comparable to the values reported by Chan and colleagues^30^ for lower prescription isodoses (5.0 ± 1.1 and 6.1 ± 1.4 for 60% and 85%, respectively) and doses to OARs are far below the constraints reported in literature.

Lastly, we would like to underline the importance of the dosimetric verification to assess proper plans deliverability. Modern planning optimization systems allow fast and efficient calculation, and planners, due to the improvement in their skills on inverse planning, tend to produce plans with high degree of modulation. Although not statistically significant, a slight degradation in the γ passing rate of the MFS plans was demonstrated in our study, while the increase in modulation was not accompanied by an improvement in the dose distribution.

## 5 CONCLUSIONS

This study describes an approach for automating the planning process using a robust template and the MCO option of Monaco TPS and demonstrates its feasibility for lung VMAT SBRT. The automatic plans are produced in a time‐efficient manner and show a high conformity and less sensitivity to interplanners variability compared to manual plans.

## CONFLICT OF INTEREST

RP is an employee of Elekta AB (Stockholm, Sweden). All other authors have no conflict of interest.

## Supporting information


**Table S1**. Cost Function and Parameters in the final automatic template. If a “shrink margin” is specified for a cost function, it means the optimizer is working on the cost function only for the voxels far from the PTV at least the distance specified by the shrink margin.

**Data S1**. Monaco (version 5.10) robust template for the 55Gy/5fr lung SBRT plan.
