# Supplementary material for: Automated planning through robust templates and multicriterial optimization for lung VMAT SBRT of lung lesions
Source: J Appl Clin Med Phys. 2020 Apr 10;21(6):114–20. doi: 10.1002/acm2.12872 (PMC7324702; doi:10.1002/acm2.12872)
Supplement: Supplementary file 1 — Table S1. Cost Function and Parameters in the final automatic template. If a “shrink margin” is specified for a cost function, it means the optimizer is working on the cost function only for the voxels far from the PTV at least the distance specified by the shrink margin. [file ACM2-21-114-s001.docx]

**Supplementary Table 1**. Cost Function and Parameters in the final automatic template. If a “shrink margin” is specified for a cost function, it means the optimizer is working on

the cost function only for the voxels far from the PTV at least the distance specified by the shrink margin.

| **Organ** | **Cost Function** | **Parameters** |
| --- | --- | --- |
| PTV | Target Penalty | Prescription (Gy) = 55  Minimum Volume (%) = 98 |
| Ring (4 cm) | Quadratic Overdose | Maximum dose (Gy) = 54  RMS Dose Excess (Gy) = 0.4 |
|  | Quadratic Overdose | Maximum dose (Gy) = 27.5  RMS Dose Excess (Gy) = 0.2  Shrink margin (cm) = 1.5 |
| Spinal Cord | Serial | Equivalent Uniform Dose (Gy) = 12  Power Law Exponent = 14  Optimize over all voxels in volume |
| Esophagus | Serial | Equivalent Uniform Dose (Gy) = 20  Power Law Exponent = 2  Optimize over all voxels in volume |
| Heart | Serial | Equivalent Uniform Dose (Gy) = 12  Power Law Exponent = 12 |
| Lungs | Parallel | Reference Dose (Gy) = 20  Mean Organ Damage (%) = 10  Power Law Exponent = 3  Optimize over all voxels in volume |
| Patient | Conformality | Relative Isoconstraint= 0.75  Optimize over all voxels in volume |
|  | Maximum Dose | 60 Gy  Optimize over all voxels in volume |
